# Evidence of the use of soft footwear in the Gravettian cave of Cussac (Dordogne, France)

**DOI:** 10.1038/s41598-021-02127-z

**Published:** 2021-11-23

**Authors:** Lysianna Ledoux, Gilles Berillon, Nathalie Fourment, Xavier Muth, Jacques Jaubert

**Affiliations:** 1 Instituto International de Investigaciones Prehistóricas Cantabria, Universidad de Cantabria (IIIPC), Edificio Interfacultativo, Avda. de los Castros, s/n, 39005 Santander, Cantabria Spain; 2 UMR7194 MNHN–CNRS/Département Homme et Environnement, Musée de l’Homme, Palais de Chaillot, 75016 Paris, France; 3 Musée National de Préhistoire, 1, Rue du Musée, 24620 Les Eyzies, France; 4 Get In Situ, Place R.T. Bosshard 1, 1097 Riex, Switzerland; 5 Université de Bordeaux, PACEA UMR 5199, B2, Avenue Geoffroy Saint-Hilaire, 33615 Pessac Cedex, France

**Keywords:** Anthropology, Archaeology

## Abstract

Humans appear to have regularly worn footwear since at least the Early Upper Palaeolithic. However, due to the perishable nature of footwear, the archaeological record of its presence during the Pleistocene is poor. While footwear would have played an essential role in protecting the foot, it could also have been used as ornamentation and/or as a social marker. Footprints may provide the most relevant insight regarding the origin and function of footwear. Here we report the discovery of footprints in Cussac Cave (southwest France) at 28–31 ka cal BP and the results of a multi-focal approach, including experimentation, that demonstrate that Gravettian people most likely wore footwear while moving through the cave. These singular footprints would constitute one of the oldest cases of indirect evidence for this unusual practice in decorated Palaeolithic caves and reinforce the exceptional nature of Cussac already attested by the presence of monumental engravings and funerary deposits.

## Introduction

The use of footwear is one element of the debate on the origins of clothing. For Pleistocene populations, footwear may have had several functions. While its most pragmatic function would have been to protect the foot from cold temperatures and the ground surface, it may also have played a more symbolic role. Ethnographic data has shown that clothing and footwear can be used as ornamentation and/or social markers^1,2^. Unfortunately, because these fragile perishable remains are rarely found in the Pleistocene archaeological record, this practice is difficult to demonstrate. Our current knowledge is based on indirect archaeological evidence associated with hide working (lithic [drill, borer] and bone tools, including needles)^3^ and artistic expressions, such as the Gravettian figurines testifying to the use of textiles^4^ or one of the most compelling examples, the Middle Upper Palaeolithic Mal’ta venus^4^. Some textile imprints in clay have also been found at the site of Dolní Věstonice (31–30 ka cal BP) and Pavlov (28–29 ka cal BP).

Nevertheless, few of these elements can be directly associated with the use of footwear^4^. The most suggestive evidence of footwear is the arrangements of beads around the feet of interred individuals at Sunghir (Russia), suggesting that they were buried with shoes^5^. Furthermore, anatomo-functional studies of modern human phalanges suggest that footwear appeared during the late Pleistocene^6,7^, though no direct evidence of footwear has been found for these periods. The oldest direct evidence of a leather moccasin-type shoe was found in the cave of Areni-1 (Armenia) and dates only to the Chalcolithic (3627–3377 cal BC)^8^. This question is rarely discussed in ichnology, probably because the great majority of recent studies are focused on barefoot tracks at Pleistocene African and European open-air sites^9–20^. Palaeolithic caves have also long been studied from this perspective due to the presence of barefoot prints in several of them^21–28^ (SI Table S1). It is in this context, in the Wahl Gallery of the Fontanet Cave (France), dated to the Magdalenian (15,076–15,398 cal BP)^29^, that the question of footwear was first raised on the basis of a footprint^29^. However, this interpretation has recently been questioned^26,30^. Dated from the Middle Palaeolithic the footprints found in the Theopetra Cave (Greece) also seem to be resulting from the use of footwear^31^. The discovery of Cussac Cave in 2000 and its systematic study since 2009, which has yielded a unique ichnological corpus associated with Gravettian people, provides new insights into this question and the understanding of human behaviour in caves.

## Contextual setting

**Figure 1:**
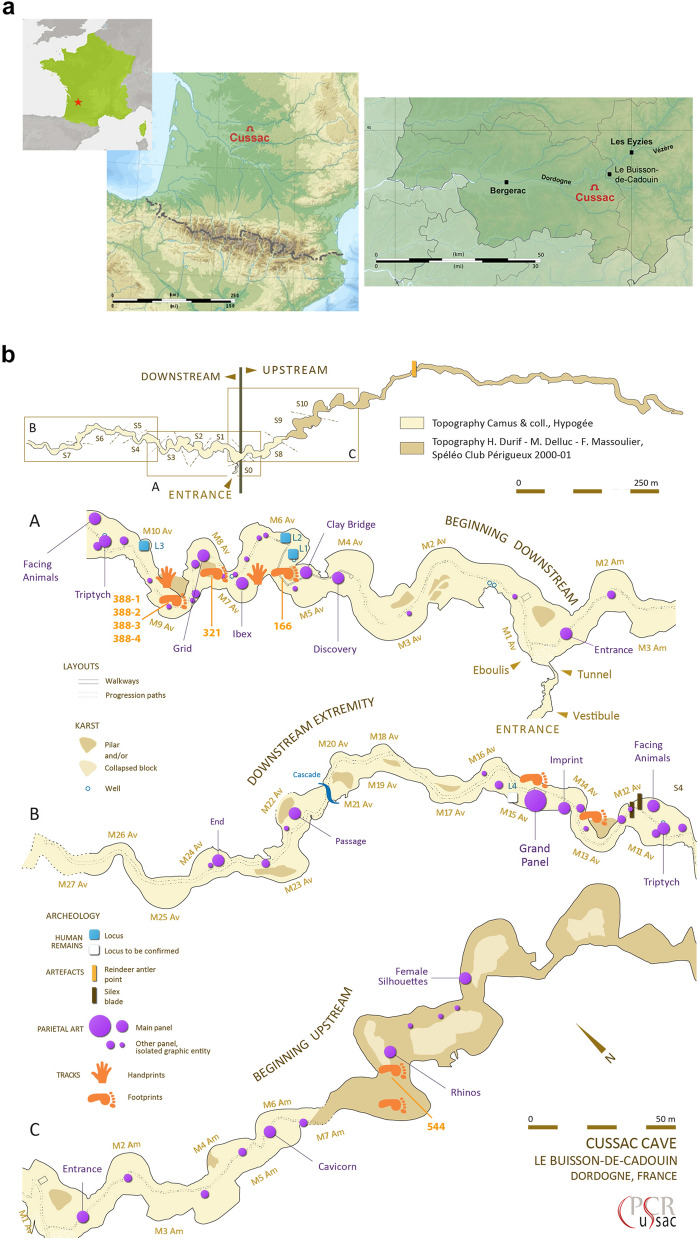
Location and topography of Cussac cave. (**a**) Geographic location of Cussac Cave. (**b**) General topography of Cussac Cave (PCR Cussac, doc. H. Camus, Protée, CAD Fr. Lacrampe-Cuyaubère) and location of the tracks (generated in Illustrator v.16.0.0, https://www.adobe.com/fr/products/illustrator.html).

**Figure 2:**
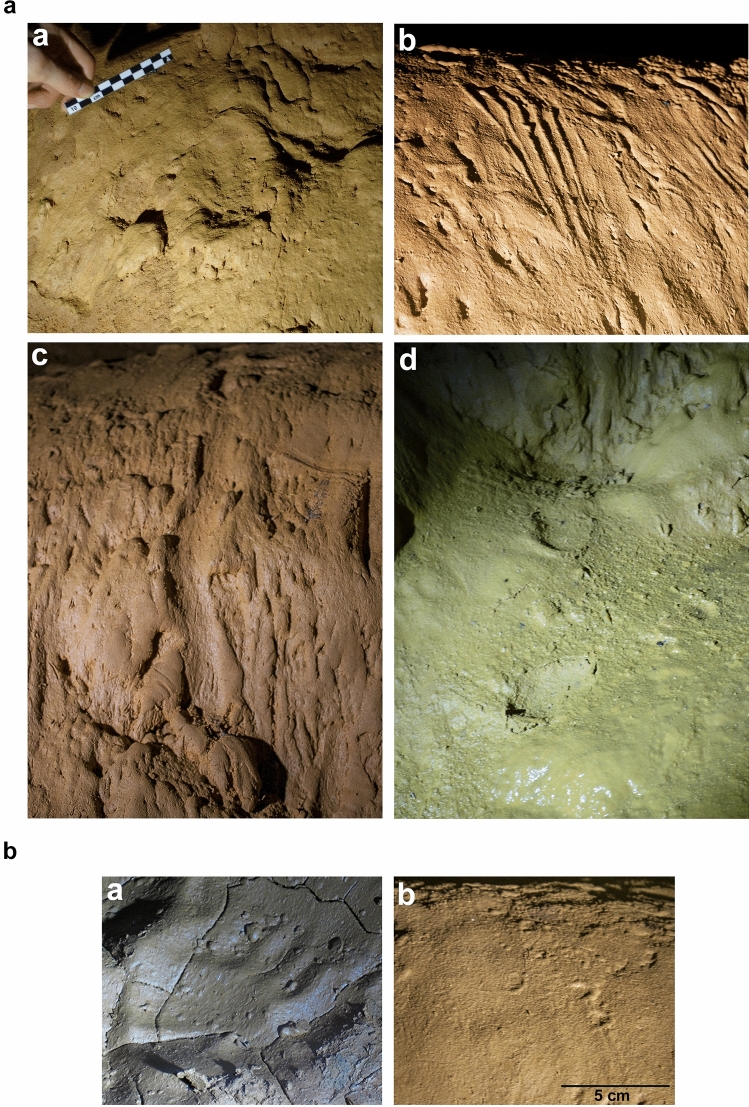
(**a**) Examples of the tracks found in Cussac Cave. (**a**) Human palm print; (**b**) indeterminate print (fingerprints?) (F. Maksud, PCR Cussac, Ministère de la Culture, France); (**c**) slipped heel print; (**d**) two heel prints belonging to a trackway. (N. Fourment, PCR Cussac, Ministère de la Culture, France). (**b**) Digit details. (**a**) Manus bear track located in the same area as T166. (**a**) Human fingerprints located under the Ibex Panel (F. Maksud, PCR Cussac, Ministère de la Culture, France ).

Located in Le Buisson-de-Cadouin (Dordogne, France), on the right bank of the Belingou (a tributary of the left bank of the Dordogne River (Fig. 1a), Cussac Cave extends over 1.6 km in a single sub-horizontal gallery. The present entrance is located nearly half-way along this gallery, making access possible in either the upstream (*Branche Amont*) or downstream (*Branche Aval*) direction of the subterranean river (Fig. 1b). This cave is characterized by monumental engravings associated with human remains deposited in bear hibernation nests as well as human and non-human activity traces (torch marks, colour marks, black or red marks, broken concretions, lamps), including prints on the ground^32–34^. Currently, after surveying only about half of the cave, 28 prints have been identified as clearly human. They correspond to complete or incomplete, deep or superficial footprints, handprints, slides and undeterminated tracks^30,33^ (Fig. 2a). The well-preserved tracks are mostly located on clay taluses, sometimes next to engraved panels, where they are protected from sedimentary deposits. Others in lower areas were covered by decantation clay^30^.

The human presence in the cave has been attributed to the Middle Gravettian. All human evidence (human bones and charcoal) on the floor and wall have been dated to 28–31,000 cal BP^35^. These absolute dates are coherent with the artistic conventions of the engravings, typical of this period, and the lithic and bone technology^36,37^. Marks made by bears always precede human marks and there is no evidence of human presence after this period. Even if the nature of the human skeletal deposits at Cussac is unique, the Gravettian period is already well-known for its burials at several European sites, in contrast to the preceding (Aurignacian) or succeeding (Solutrean) periods. Therefore, the presence of early human footprints in this exceptional context and their study is particularly challenging.

Here, we present the seven complete footprints thus far identified in the cave. These footprints display the main morphological characteristics of human footprints. However, while the area of the digits is well preserved, the digit impression is shallow or absent. This phenomenon is particularly remarkable because in the same cave areas, well-marked fingerprints are present (Fig. 2b), whereas toe prints are always missing. In these cave areas, the footprints consist of partially hollowed spaces that are difficult to characterize. Furthermore, when bear and human tracks are superimposed, the digits of the complete human footprints are absent, whereas the digits are clearly visible on the bear tracks (Fig. 2b). Finally, although flooding was recurrent in some parts of the cave, we have demonstrated through experimentation^38^ that this phenomenon alone cannot explain the disappearance of the footprint digits. While the print made by footwear is most likely related to the nature of the footwear itself, it is also determined by the substrate. This footwear-substrate combination has never been quantified in this type of context, however. In this study, we thus test the hypothesis of the use of footwear by the Gravettian people at Cussac by experimentally quantifying the morphology of the footprints made by soft footwear on a clay substrate similar to that of Cussac.

## Results

**Figure 3:**
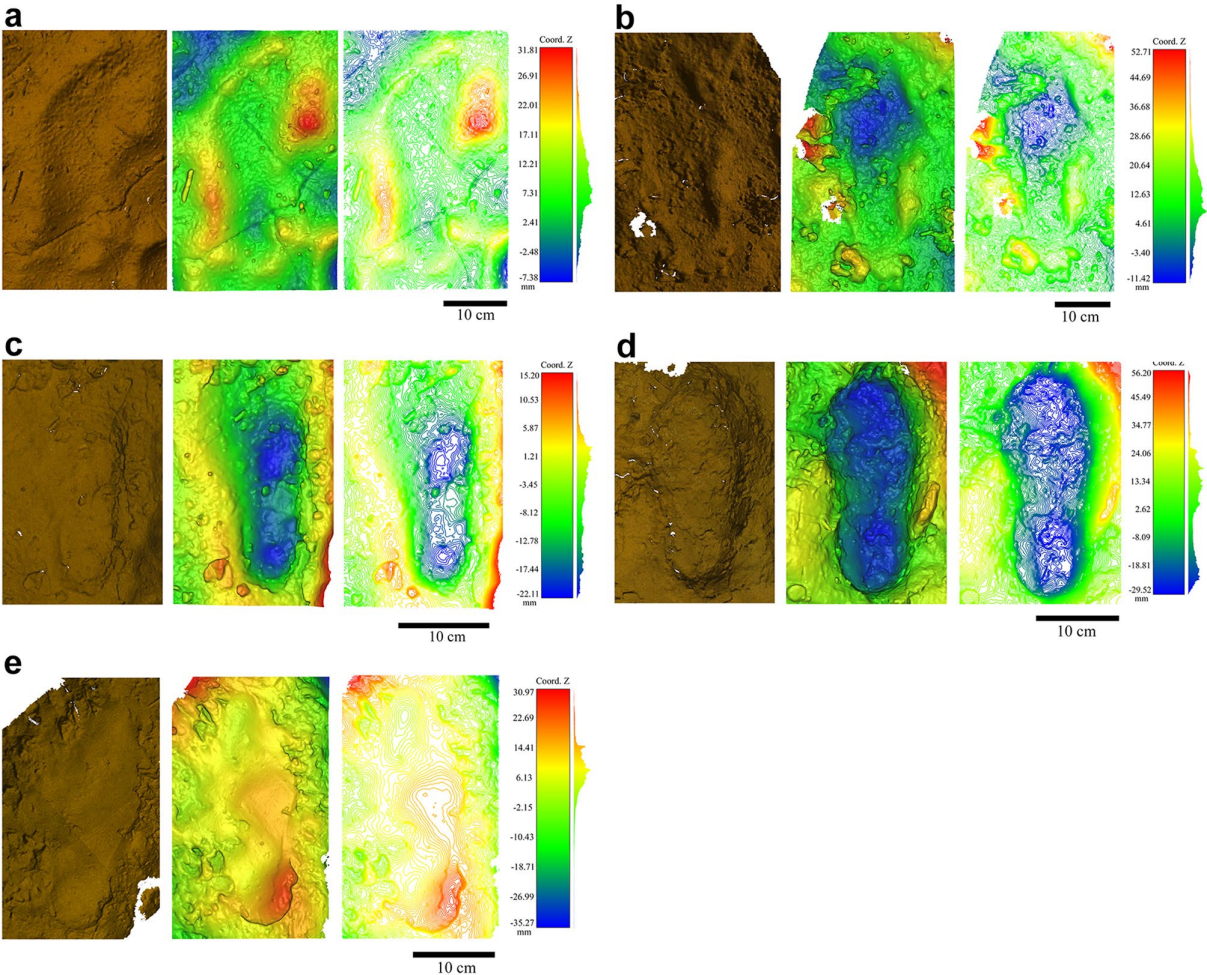
True colour images, depth maps and contour maps of the Cussac footprints. (**a**) T166; (**b**) T321; (**c**) T388-1; (**d**) T388-4; (**e**) T544.

Among the seven complete footprints, three are isolated (T166, T321, T544), and four form a partial trackway. Two of them are relatively well-preserved (T388-1 and T388-4), while recent trampling has significantly altered the other two (T388-2 and T388-3) (Fig. 3, SI Fig. S1, SI Video 1). All the tracks are formed in a high clay content sediment favourable to track formation (SI Table S2). Their length ranges from 19 to 30 cm and the general morphology of the foot is preserved (Fig. 3 and Table 1). The distal and proximal parts and the medial longitudinal arch are well identifiable, allowing their lateralisation: three left feet and four right feet. Most of these footprints are relatively shallow. The last footprint of the trackway (T388-4) is deep compared to the others; its location on a small clayey bench favoured the foot’s penetration through the sediment. Although in some cases the mark of the hallux is visible, no other toe has left an impression regardless of the plasticity of the sediment.

**Table 1:** Characteristic of the Cussac Cave footprints (cm).

Footprint	Length 1 (cm)	Distal width (cm)	Middle width (cm)	Proximal width (cm)	Location	Lateralization	Sediment formation	Preservation
T 166	30.1	10	9.4	6.8	Downstream	Left	Clay	Covered with decantation clay
T 321	31	8.9	8.3	6.3	Downstream	Left	Clay	Good
T 388-1	19	4.5	5.6	5.3	Downstream	Rigth	Clay	Good
T 388-4	24.8	7.4	6.8	5.7	Downstream	Rigth	Clay	Good
T 544	26.5	7.6	5.2	5.1	Upstream	Left	Clay	Altered by modern trampling

**Figure 4:**
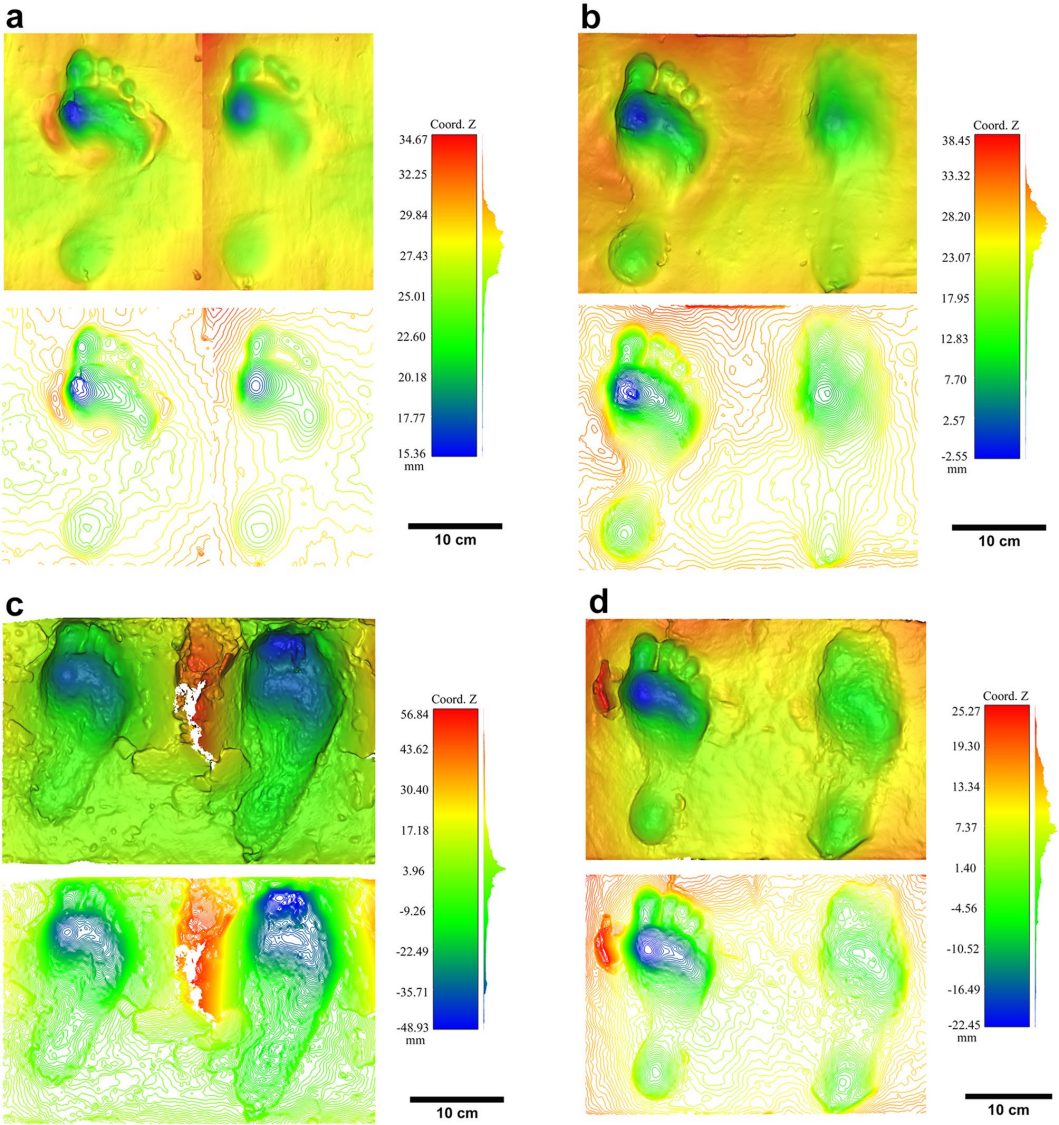
Depth maps and contour maps of some experimental barefoot prints and their associated footwear prints. (**a**) ch2 (without stuffing, moisture content of 50%); (**b**) ch6 (with stuffing, moisture content of 50%); (**c**) ch16 (without stuffing, moisture content of 60% 70%); (**d**) ch18 (stuffing, moisture content of 60% 70%).

We conducted experiments in a substrate with sedimentary properties very close to those of Cussac, with two moisture contents (SI Table S2 and “Methods”). Original barefoot prints made in this type of sediment show that whatever the moisture content, the characteristics of the general morphology of the foot are clearly visible with details such as well-marked toes. This observation is valid for the two moisture contents tested (Fig. 4). To go further, we tested the influence of soft leather footwear (with and without stuffing) on the footprint morphology using the same sediment and moisture contents. The results demonstrate that although the general morphology of the foot is preserved on the footwear tracks, the use of footwear affects their geometry and biometry. In addition, the footwear tracks tend to become longer and narrower whatever the substrate and with or without stuffing.

**Figure 5:**
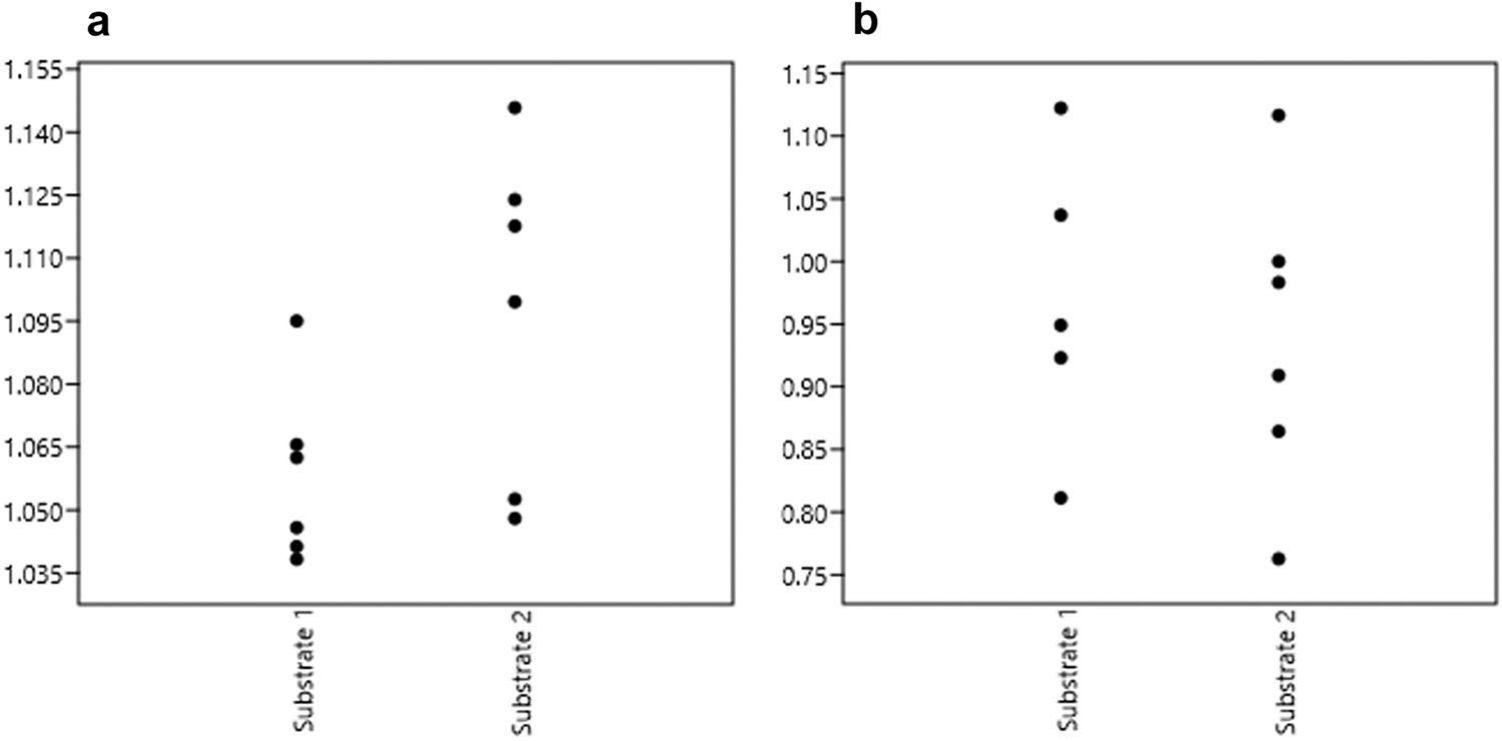
Jitter plots of ratios computed on footprints. (**a**) Ratio between the length of footwear prints and barefoot prints for the two substrate types; (**b**) ratio between the width of the proximal part of footwear prints and barefoot prints for the two substrate types.

In the same way that footprints are almost always longer than the trackmaker’s feet—especially when the substrate is compliant and the footprints are deep^39^ (Fig. 4 and SI Table S3), the ratio between the length of a footwear print and a barefoot print shows that prints made with footwear are always longer than the barefoot ones, especially when associated with the wettest substrate (Fig. 5a). However, the ratio is more variable when comparing the width of the proximal part of footprints made by footwear with that of barefoot prints (Fig. 5b). This experiment also highlights the presence of digits on the footprints made with footwear without stuffing, particularly when associated with the first substrate (50% moisture content substrate). Due to its higher position, the hallux is the only digit that can be identified on some footprints made with stuffed footwear (Fig. 4). Finally, the profiles and depth maps of our experimental tracks show that almost all the footwear prints appear shallower than the barefoot prints, especially in the distal and proximal parts.

**Figure 6:**
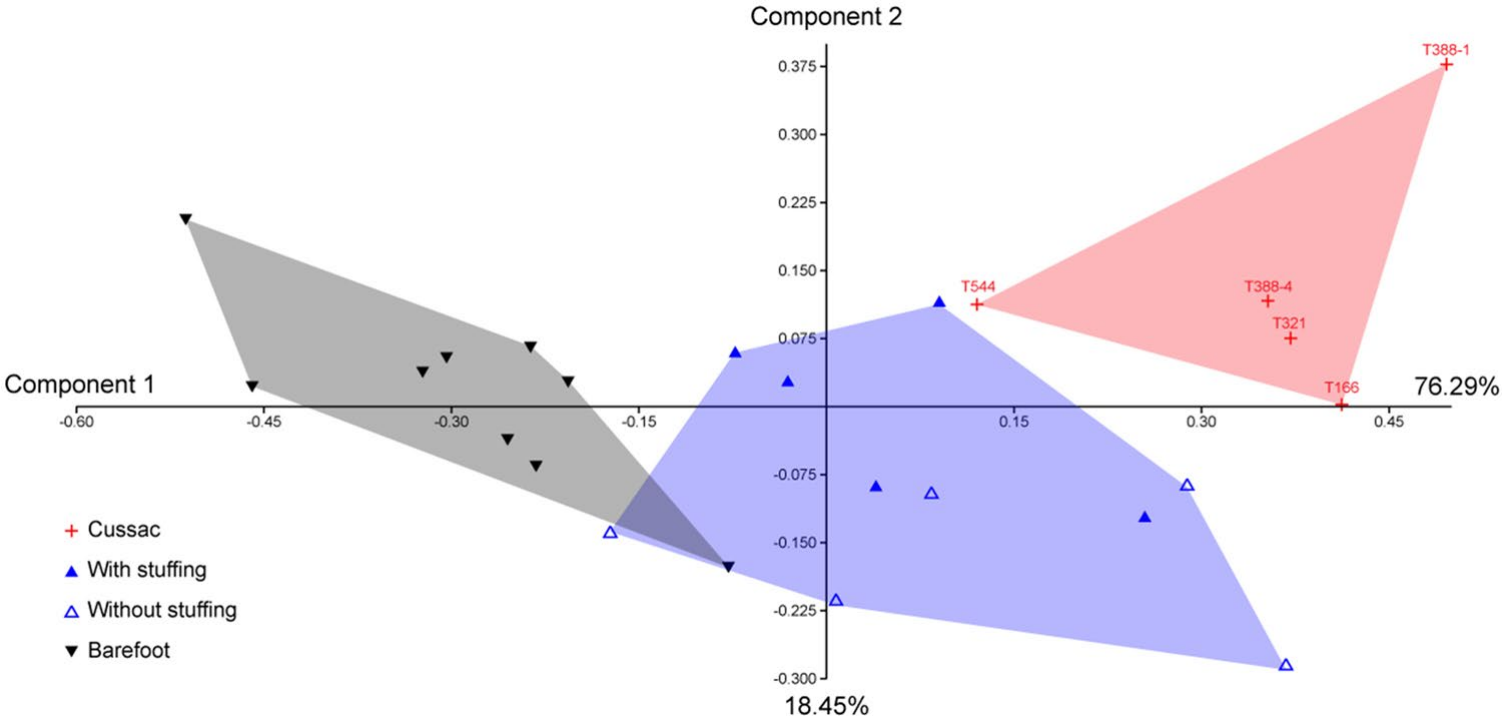
Principal component analysis performed on log-transformed data of Cussac and experimental footprints.

We used the *log-shape ratio* methodology to compare the shape of the best-preserved footprints of Cussac and some experimental footprints (SI Table S4). A global PCA was performed on log-transformed data. The first two principal components explain 94.74% of the variance. While length 1 and distal width drives both axes almost equally, the first axis (76.29% of the variance) is mainly driven by the medium width. Along this axis, the Cussac footprints are projected opposite to the experimental barefoot prints and close to the experimental footwear prints; these footwear prints form an intermediate and distinct but more variable group. The second axis (18.45% of the variance) is driven by the proximal width. Along this axis, the three experimental footwear prints located in the upper part of the cloud correspond to the driest clay. Interestingly, the Cussac footprint T544, formed in the most cohesive and firm substrate, projects close to these three tracks. The footprints T388-4, T321, and T166, constitute an isolated cluster close to some experimental footprints (Fig. 6).

In summary, despite inter-individual differences, the results of our multi-focal approach confirm that a firm and cohesive substrate favours the impression of details, especially in the distal foot area where the toes create a complex geometry. Moreover, in two distinct moisture contents, footwear impacts the geometry of the footprint specifically in this distal part. When covered with footwear, the distal portion of the foot becomes simpler and more homogeneous (compared to the complex shape formed by the separated toes of a bare foot) and, consequently, the toes are less visible. Similarly, the use of a footwear increases the medium width and simplifies the shape of the print by modifying the impression of the plantar arch. This simplification of morphology also impacts the depth of the footwear prints being shallower than barefoot prints. Therefore, the results of our multi-focal approach converge toward the conclusion that the hypothesis of footwear use is the one that most likely corresponds to the morphology of the Cussac footprints. In this vein, it is significant that, despite a detailed and extensive survey of Cussac Cave, very few footprints have been identified, even under the decorated panels, where most of the activities in the cave are concentrated. The use of footwear can also explain this peculiarity of the Cussac floor.

## Discussion

Our study of the footprints in Cussac Cave revisits the debate on the origin of footwear and raises new questions about the function of both footwear and that of Palaeolithic caves. In Cussac Cave, the presence within the same gallery of monumental engravings and human deposits reflects its singular nature. This uniqueness is reinforced by the originality of its footprints suggesting the use of footwear inside the cave. Thus, this would be an original behaviour as the footprints found in caves are generally known to be barefoot prints^21–25,27,28,40–43^. Is this specificity of Cussac due to a problem of differential preservation or taphonomy in other sites? Or simply to our ability to identify shod footprints, which are less evident than barefoot prints? If the individuals in Cussac Cave indeed wore footwear, does this reflect a specific function of this cave? Is this function necessarily related to the sepulchral dimension of the cave? Or does it reflect a more practical aspect? Studies are still in progress, these questions currently remain unanswered. However, it is certain that the human remains form part of a coherent whole reinforced by the homogeneity of the dating already carried out. All the evidence suggests a minimal number of incursions, maybe one generation of human groups. Meanwhile, we cannot affirm that the bodies deposited in the bear nests and the individuals who deposited them belonged to the same group^44^ although this is the most parsimonious hypothesis in the current state of analysis. Nevertheless, the study of foot bones found in the cave will also shed new light on the question of footwear. However, as is usual for this type of site, the national heritage status of Cussac Cave limits access to the human remains, which are still inventoried and studied but cannot be excavated. It is nevertheless possible that someday we will be able to link the bone remains to footprints. This discovery and study of footprints in Cussac Cave already contributes new information about footwear, for which there is no direct evidence in these periods. It also sheds new light on a specific practice within the restricted space of caves and, more generally, at Palaeolithic societies. Whether footwear was used to protect the feet or as ornamentation, its use in Cussac Cave would be among the earliest indirect evidence of this practice by Pleistocene populations.

## Methods

Our study is based on seven complete footprints identified in Cussac Cave. These footprints were discovered during the survey and inventory of human and animal activity traces, ongoing since 2009^33^.

Experiments were performed using a substrate favourable to the impression of the foot and similar to the substrates of Cussac. They were carried out at the Pôle Mixte de Recherche Archéologique of Campagne and at the PACEA laboratory of the University of Bordeaux (France). The footprints were made in boxes of identical dimensions: 50 × 40 × 25 cm.

The experimental footprints were thus made in a cohesive and moist sediment with a high clay content. This sediment was collected from a cave in the Dordogne region of southwestern France, containing no archaeological remains. Its sedimentary properties are close to those of Cussac (SI Table S2). From this sediment, we tested a moisture content of approximately 50%, and a moisture content varying between 60 and 70%.

The granulometric analysis was performed at the PACEA laboratory using Horiba LA-950 laser diffraction particle size distribution analysers.

The moisture content was calculated on a wet-weight basis using the following formula:

$$\left(\frac{m1-m2}{m1-m0}\right)\times 100$$

where, m0 is the weight of container with lid, m1 is the weight of container with sample and sample before drying, m2 is the weight of container with sample and sample after drying.

We used shoes based on those of Areni-1 and the Ötzi mummy^8^. They were made from a single piece of hide leather, approximately 2 mm thick. The leather was wrapped around the foot and attached with a leather strap (SI Fig. S2). In some cases, straw-based stuffing was added.

The experimental footprints were made by three people: a female (Individual 1), 1.69-m-tall, weighing 55 kg, with a foot length of 24 cm; a male (Individual 2), 1.80-m-tall, weighing 80 kg, with a foot length of 24 cm, and; a male (Individual 3), 1.68-m-tall, weighing 75 kg, with a foot length of 23.5 cm. Ethical approval was granted by the PACEA laboratory of the University of Bordeaux (France). All individuals gave informed consent and experiments were conducted in accordance with the American Psychological Association (APA) Ethics Code.

Each individual made four footwear prints. The controlled parameters are the characteristics of the shoe and the moisture content of the substrate. The combinations used are therefore:

- shoe without stuffing + moisture content of 50%
- shoe with stuffing + moisture content of 50%
- shoe without stuffing + moisture content between 60 and 70%
- shoe with stuffing + moisture content between 60 and 70%

For the comparative analysis, each footwear print was associated with a barefoot print (SI Fig. S3). A total of 24 footprints were made (12 footwear prints and 12 bare footprints).

All the footprints (archaeological and experimental) were described in detail and a maximum of seven measurements considered as most indicative of the print morphology were recorded: length 1 (distance between the most distal point of the hallux and the most inferior point of the pternion); length 2 (distance between the most distal point of the second toe and the most inferior point of the pternion); length 3 (distance between the most distal point of the forefoot and the most inferior point of the pternion); digit width (distance between the most medial point of the hallux and the most lateral point of the last toe); distal width (distance between the most medial point and the most lateral point of the forefoot); middle width (distance between the most medial point and the most lateral point of the longitudinal arch), and; proximal width (distance between the most medial point and the most lateral point of the heel). Length 2, length 3, and digit measurements were not recorded because the Cussac footprints’ and most of the experimental footwear prints do not have visible digits.

The footprints were photographed using a Nikon D7100 with a 60 mm focal length lens. Each footprint was digitized in 3D using an Artec EVA 3D light scanner (2013; Artec Group, Luxembourg). This device uses the structured light triangulation technique to reconstruct a 3D model of the footprint. The accuracy achieved by this scanner is 0.5 mm at a working distance of 40 cm–1 m and the 3D resolution is up to 0.1 mm. The scanner takes up to 16 frames per second and passes them over to the Artec Studio software^45^, which aligns the frames in real time.

Post-processing was performed on the Artec Studio 9 software to recreate a colour texturized 3D mesh.

The 3D models of the footprints were visualized and compared with Cloud Compare (2.8.1.). We used part of the standard protocol proposed by Falkingham et al.^46^ to record, present and archive our 3D data. True colour images, depth maps and contour maps (range of 0.5 mm) were therefore created for each footprint.

A multivariate analysis on transformed data was performed to compare fossil footprints and 19 of the 24 experimental footprints (9 footwear prints and 10 bare footprints). In order to limit the effect of size and consider mostly the shape of the footprints, log-shape ratios were calculated based on the raw linear dimensions of the footprints. A Principal Component Analysis (PCA) was then performed on the log-shape ratios to visualize difference between barefoot print and footwear prints.

## Supplementary Information

Supplementary Information.

Supplementary Video 1.

## Data Availability

The datasets generated during and analysed during the current study are available from the corresponding author on reasonable request.
