# Supplementary material for: Evidence of the use of soft footwear in the Gravettian cave of Cussac (Dordogne, France)
Source: Sci Rep. 2021 Nov 23;11:22727. doi: 10.1038/s41598-021-02127-z (PMC8610977; doi:10.1038/s41598-021-02127-z)

**Evidence of the use of soft footwear in the Gravettian cave of Cussac (Dordogne, France)**

Lysianna Ledoux, Gilles Berillon, Nathalie Fourment, Xavier Muth, Jacques Jaubert

## Supplementary Information

**Table S1:** Palaeolithic decorated caves that have preserved footprints[1]. Most of the time they are complete or incomplete foot or hand prints.

| Cave | Age | Type |
|---|---|---|
| Aitzbitarte (Spain) *Unpublished* | - | Handprint |
| Atxurra (Spain) *Unpublished* | - | Handprints, footprints ? |
| Chauvet (France)[1,2] | 37,000 -28,500 cal BP | Footprints |
| Les Combarelles III [3](France) | 13,680 BP-24,500 BP | Handprint |
| Cournazac (France) *Unpublished* | - | Fingerprints |
| Cussac (France)[4] | 28-31,000 cal BP | All kind of tracks |
| El Castillo (Spain)[5] | 34,058 ± 244 -<br>28,882 ± 391 cal BP | Footprints |
| Fontanet - Réseau Wahl (France)[4] | 12,770 ± 42 BP | All kind of tracks |
| La Garma (Spain) *Unpublished* | - | Footprints |
| Lascaux (France)[6] | - | Handprint, footprints |
| Montespan (France)[7] | - | Footprints |
| Niaux - Réseau Clastres (France)[8–10] | ≈ 12,000 BP | Footprints |
| Ojo Guareña (Spain)[11] | 15,600 BP | Footprints |
| Pech Merle (France)[12,13] | 24,640 ± 390 cal BP | Footprints |
| Tuc d'Audoubert (France)[10] | 13,870 BP | Footprints |

Despite the existence of nearly 400 decorated Palaeolithic caves in Europe, very few of them contain prints. One of the possible explanations for this is that many early discoveries were made before research included considerations of the ground, taphonomic problems destroying the prints or observation bias. Not all the footprints currently recorded in Palaeolithic caves can be studied. Some of them are incomplete, poorly preserved or isolated. Most existing studies concern footprints composing trackways, which provide information on the biology, locomotion, behaviour and activities of the trackmakers in the cave. The Cussac Cave footprints are particularly significant in this corpus because they provide information on an unprecedented practice in decorated caves.

1. Garcia, M.-A. Ichnologie générale de la grotte Chauvet. *Bulletin de la Société préhistorique française* **102**, 103–108 (2005).

2. Quiles, A. *et al.* A high-precision chronological model for the decorated Upper Paleolithic cave of Chauvet-Pont d'Arc, Ardèche, France. *Proceedings of the National Academy of Sciences* **113**, 4670–4675 (2016).

[1] It should nevertheless be noted that the footprints of the Aldène and Ojo Guareña are more recent and not associated with the Palaeolithic art of the caves.

3. Cleyet-Merle, J.-J., Feruglio, V. & Delluc, M. Combarelles III (Les Eyzies-de-Tayac, Dordogne, France). *PALEO Revue d'Archéologie Préhistorique* 115–128 (2016).

4. Ledoux, L. L'ichnologie préhistorique et les traces d'activités au sein des cavités ornées. Les grottes de Fontanet (Ariège) et de Cussac (Dordogne). (Université de Bordeaux, 2019).

5. Groenen, M. Bilan de sept années de recherches dans la grotte ornée d'El Castillo (Cantabrie, Espagne). in *Actes du Congrés IFRAO, septembre 2010 - Symposium ' Art pléistocène en Europe'.* 145–163 (Clottes, J. (dir.), 2012).

6. Barrière, C. & Sahly, A. Les empreintes humaines de Lascaux. in *Miscelánea en homenaje al abate Henri Breuil 1877-1961* 173–180 (Instituto de Prehistoria y Arqueologia, 1964).

7. Bégouën, H. & Vallois, H. V. *Les empreintes de pieds préhistoriques.* (E. Nourry, 1928).

8. Pales, L. *Les Empreintes de pieds humains dans les cavernes . Les empreintes du réseau nord de la caverne de Niaux (Ariège)*. (1976).

9. Garcia, M.-A., Duday, H. & Courtaud, P. Les empreintes du Réseau Clastres. *Préhistoire Ariégeoise* **45**, 167–174 (1990).

10. Pastoors, A. *et al.* Tracking in Caves: Experience Based Reading of Pleistocene Human Footprints in French Caves. *Cambridge Archaeological Journal* **25**, 551–564 (2015).

11. Ortega Martinez, A. I. *et al.* Escaneado en 3D de las Galerías de las Huellas (Ojo Guareña, Merindad de Sotoscueva, Burgos). *Cubía* 38–47 (2014).

12. Pastoors, A. *et al.* Experience based reading of Pleistocene human footprints in Pech-Merle. *Quaternary International* **430**, 155–162 (2017).

13. Duday, H. & Garcia, M. A. Les empreintes de l'Homme préhistorique. La grotte du Pech-Merle à Cabrerets (Lot): une relecture significative des traces de pieds humains. *Bulletin de la Société préhistorique française* 208–215 (1983).

**Table S2:** Grain size analyses of the sediment sampled in Cussac cave and in the experimental cave.

| Grain Size Analyses | | | | |
|---|---|---|---|---|
| Samples | % fine sand (500-63 µ) | % coarse silt (63-16 µ) | % fine silt (16-7 µ) | % clay (<7 µ) |
| Cussac | 2.56 | 18.99 | 22.59 | 55.85 |
| Experimental cave | 2.8 | 10.78 | 30.42 | 56 |

**Table S3:** Biometry of the experimental footprints. White boxes: barefoot; Light grey boxes: shoe without stuffing; Dark grey boxes: shoe with stuffing (cm).

| Moisture content | Footprint | Length 1 | Distal width | Middle width | Proximal width |
|---|---|---|---|---|---|
| 50% | ch1 | 24.4 | 8 | none | 4.3 |
| | ch1 | 23.5 | 8.2 | 2.5 | 5.3 |
| | ch2 | 25.2 | 9.6 | none | 4.8 |
| | ch2 | 24 | 10 | none | 5.2 |
| | ch11 | 25.5 | 9.6 | none | none |
| | ch11 | 24 | 9.4 | 3.4 | 52 |
| | ch4 | 25.2 | 8 | 5.2 | 5.6 |
| | ch4 | 24.2 | 8.1 | none | 5.4 |
| | ch6 | 26.5 | 9.4 | 5 | 5.6 |
| | ch6 | 24.2 | 10 | 3.7 | 5.9 |
| | ch12 | 26 | 9 | 4.6 | 5.5 |
| | ch12 | 24.4 | 10 | 3.9 | 4.9 |
| 60%/70% | ch3 | 26.6 | 10.4 | 4.3 | 4.5 |
| | ch3 | 23.8 | 9.6 | 3.9 | 5.9 |
| | ch13 | 28 | 13.1 | 6.5 | 5.9 |
| | ch13 | 26.6 | 11.4 | 3.4 | 6 |
| | ch16 | 27.5 | 12 | 6.9 | 6.7 |
| | ch16 | 24 | 12.4 | 5.5 | 6 |
| | ch10 | 26.3 | 9.9 | 5.5 | 5.1 |
| | ch10 | 23.4 | 9.9 | 4.1 | 5.9 |
| | ch14 | 26.2 | 10.6 | 7.6 | 5.9 |
| | ch14 | 25 | 11.1 | 4.2 | 5.9 |
| | ch17 | 29.2 | 12.9 | 10 | 5.9 |
| | ch17 | 24.6 | 10.5 | 7.9 | 6.5 |

**Table S4:** Log-transformed data of Cussac and experimental footprints used for the Principal component analysis. White boxes: barefoot; Light grey boxes: shoe without stuffing; Dark grey boxes: shoe with stuffing.

| Footprint | Length 1 | Distal width | Middle width. | Proximal width |
|---|---|---|---|---|
| T166 | 0.93833953 | -0.16360055 | -0.22547595 | -0.549263029 |
| T321 | 1.0397763 | -0.20815963 | -0.27795539 | -0.553661274 |
| T388-1 | 0.98469153 | -0.45567005 | -0.23698085 | -0.292040628 |
| T388-4 | 0.99341554 | -0.21594811 | -0.3005055 | -0.476961935 |
| T544 | 1.1313467 | -0.11764979 | -0.49713941 | -0.516557497 |
| ch1 | 1.195717389 | 0.142851122 | -1.0449923 | -0.293576211 |
| ch11 | 1.105254436 | 0.167910295 | -0.849023963 | -0.424140769 |
| ch4 | 1.057416305 | -0.089986148 | -0.520769064 | -0.446661092 |
| ch6 | 1.06463 | 0.028194956 | -0.603076821 | -0.489748135 |
| ch6 | 1.043296909 | 0.159529369 | -0.834722905 | -0.368103373 |
| ch12 | 1.08656516 | 0.0256932 | -0.645475074 | -0.466783286 |
| ch12 | 1.082738136 | 0.190740097 | -0.750868443 | -0.522609791 |
| ch3 | 1.134558855 | 0.195453446 | -0.687737338 | -0.642274964 |
| ch3 | 1.027841185 | 0.119918703 | -0.780867843 | -0.366892045 |
| ch13 | 0.944311693 | 0.184719413 | -0.51609064 | -0.612940466 |
| ch13 | 1.098396348 | 0.251098487 | -0.958739436 | -0.390755399 |
| ch16 | 0.906005607 | 0.076726252 | -0.476658987 | -0.506072872 |
| ch16 | 0.879989364 | 0.219632007 | -0.593316374 | -0.506304997 |
| ch10 | 1.045545857 | 0.068511675 | -0.51927499 | -0.594782542 |
| ch10 | 0.994933496 | 0.134732231 | -0.746815552 | -0.382850175 |
| ch14 | 0.908330908 | 0.003425499 | -0.329280255 | -0.582476152 |
| ch14 | 1.009911373 | 0.197980656 | -0.773879927 | -0.434012101 |
| ch17 | 0.871935343 | 0.054993945 | -0.199648273 | -0.727281015 |
| ch17 | 0.829549784 | -0.021821402 | -0.3063339 | -0.501394482 |

**Figure S1: Photographs and descriptions of Cussac footprints.** a: T166 (L. Ledoux, PCR Cussac, Ministère de la Culture, France ©): left footprint located in the downstream part of the cavity near the area where the human remains were deposited. It was made on a flat clay surface and was covered with decantation clay resulting from several flooding episodes in the cavity. Despite this, it is complete and relatively well-preserved. Its outline is clearly visible with all the foot areas represented. Although no detail is apparent, the mark of what could be consistent with the hallux is observed. b: T321 (F. Maksud, PCR Cussac, Ministère de la Culture, France ©): left footprint located in the downstream part of the cavity in the area of the Grid Panel. It was made on a flat clay surface in a high humidity area. It is complete and its outline is quite visible with prominent raised rims at its medial and lateral edges. Although the forefoot and heel are relatively shallow, all the foot areas are represented. Digit detail is missing but a slide trace on the front of the foot appears to be consistent with the hallux mark. c: T544 (L. Ledoux, PCR Cussac, Ministère de la Culture, France ©): left footprint located in the upstream part of the cavity near the area of the Rhinos Panel. It was made against the direction of the slope of a clayey talus. Despite its shallowness, its outline is clearly visible with all the foot areas represented. A prominent raised rim is visible in the heel area. Other than the hallux mark, the front of the foot shows no details. d, e, f: T388: partial trackway located in the downstream part of the cavity and composed of four right footprints. The rest of the trackway (left footprints) was probably destroyed by the current path. The trackway was made on a flat clay surface and runs along a flowstone. Two of the footprints are relatively well preserved: d: T388-1 (N. Fourment, PCR Cussac, Ministère de la Culture, France ©) is the first footprint of the trackway. It is a shallow right and almost complete footprint with a relatively well-marked heel. The medial edge of the forefoot is absent, however. It is the best-preserved footprint of the trackway. No disturbance is observed on its surface; e: T388-4 (N. Fourment, PCR Cussac, Ministère de la Culture, France ©) is the last footprint of the trackway. It is a right deep and complete

footprint with its outline clearly visible. It has been slightly altered by modern trampling. The two other footprints have been heavily altered by modern trampling (T388-2 and T388-3). Their outlines are rather ill-defined, and they are covered with clay pellets.

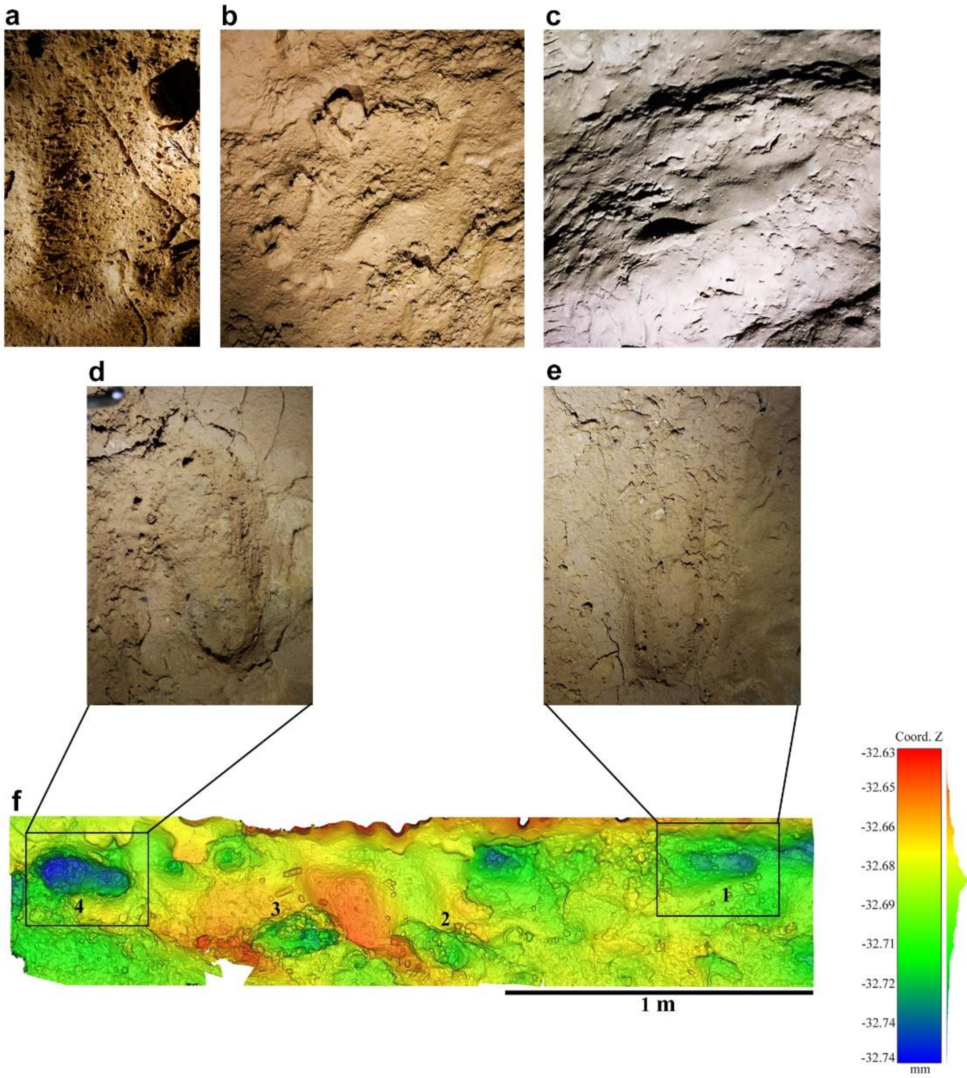

**Figure S2:** Experimental footwear made from a single piece of hide leather (M. Baillet), approximately 2-mm-thick. It was wrapped around the foot and attached with a leather strap (L. Ledoux, PCR Cussac, Ministère de la Culture, France ©).

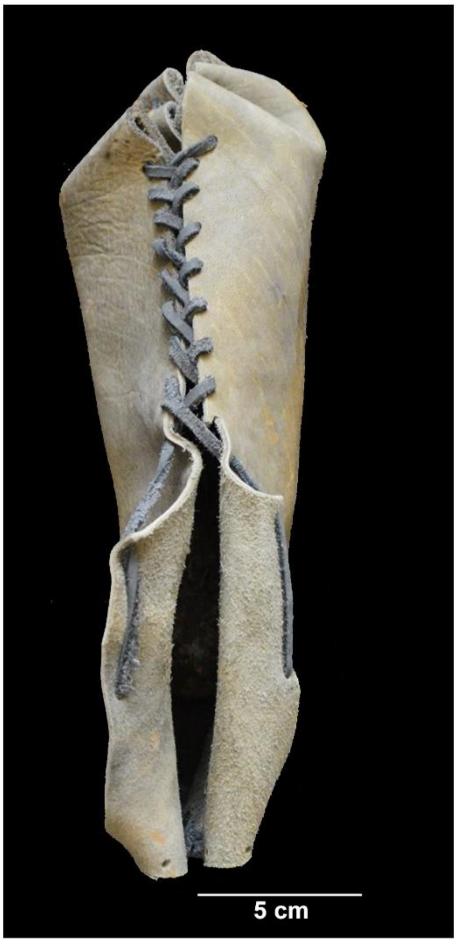

**Figure S3:** Experimental steps.

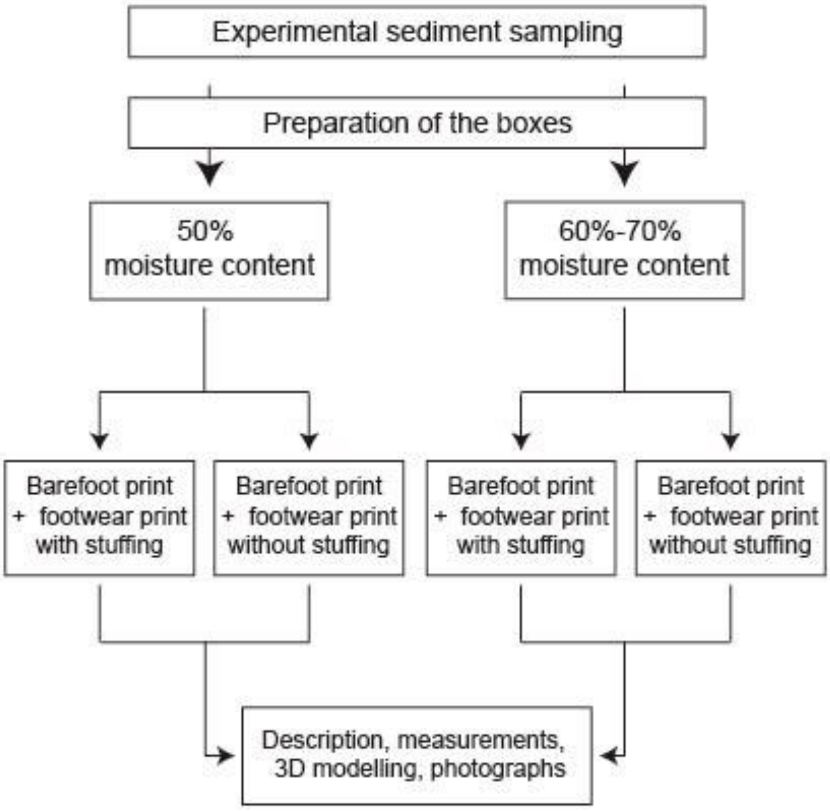

Supplement: Supplementary file 1 — Supplementary Information. [file 41598_2021_2127_MOESM1_ESM.pdf]
